# The New Title and Secession

**Published:** 1861-01

**Authors:** 


### EDITORIAL AND MISCELLANEOUS.

### The New Title and Secession.

Much has been said of late, in *Northern Journals*, about a change in the title M. D., the tendency of which is to favor the plan of making membership in the American Medical Association necessary to the right of *the new* title. Hence the term, *Member of American Medical Association*, has been proposed, the abreviated letters of which to be substituted for M. D., thus: *John Smith*, *M. A. M. A.*

We object to this innovation for several reasons: 1st. Because many learned, respectable and useful physicians, are not, never have been, nor ever will be members of the American Medical Association. And though the association should pass an ordinance declaring all such as would not apply for membership out of the profession and unworthy a position in it, there would still be hundreds of our most talented physicians left out of its pales. What would be the effect of the movement under these circumstances? Certainly to divide educated members of the profession, and leave a plausible pretext for the many sects to claim equal *regularity* with true medicine. 2d. Because the term does not imply anything at all more than the mere membership of an association, which the whole world looks upon, and justly too, as an empty sound. To be *learned in Medicine*, which M. D. is intended to represent, is to be what is required of the medical man; and we ask, will the schools of this country sign a diploma when as an examining committee, they would not admit such an applicant to membership in the association? It may be answered that the schools do not constitute the association. Granted, but who will deny that Medical Colleges make up a large proportion of the representation, and exert a controlling influence in that body?

The truth is, colleges, as well as others, have become sick and tired of finding the profession crowded in many parts of the country with physicians illy prepared for the duties of the profession; and to remedy this evil by raising the standared of green-room requirements, would drive back many 105's from their coffers. Get the money, count a large class of students, and set a trap to behead the unworthy after all the service they can render the colleges is completed, would seem to be the programme. How much better for the unsuspecting student; how much better for the profession, and the community to make the nominal requirement of *learned in Medicine*, a real one. If the association desires to suggest a remedy for the evil of granting the degree, improperly let that suggestion be made to the colleges; not as directions how they shall teach, how long or when the student must study, but that the degree of M. D. should be withheld from all who are not *learned in Medicine.*—These are reasons enough to satisfy us of the impropriety of such change, but we have others: The legitimate result of such a course will be to create an aristocracy in Medicine, not of the properly learned specially, but composed of those, to a considerable extent, who can make a show of words without being of any practical benefit to the profession or to the world. This move concentrates the power of the Association more perfectly for the oppression of what ever section or Institution the would-be lords of the profession may find it to their interest to grind. The powers and influences of the body, are under the present organization, too easily directed against those sections whose representation happens to be in the minority. Under the semblance of some great good to the profession these attacks are made. Such was the pretext last June in New Haven, when a Summer Institution in Georgia, the Atlanta Medical College, was indirectly attacked, under the ostensible object of improving the system of medical education. This attack was threatened on several occasions, when the convention met South, but though some covert wire pullers might gladly have aided in such a measure, the mass of the profession at home, frowned upon it, and a large majority of the representation in attendance upon Southern meetings of the body, strangled it in its inception. Not so in Connecticut. Other feelings, other motives and other principles governed the convention there, than those that prevailed in Nashville, Washington and Louisvillle, because the controlling representation coming from the section of the Union in which the meeting occured, opposed us. This antipodal feeling permeates every branch and ramification of society—religious political and medical. In New Haven the representation was unusually sectional. Very few representatives South of Mason and Dixon's line. Hence the suggestion of a summer school of medicine in Georgia likely to prosper, and the idea of working its destruction under the name, and by mounting the hobby of improvement in medical education, was sufficient for the work to be done. And what was the improvement in the system of medical education made? Was it that students should be thoroughly educated before the degree is conferred? No, nothing of the kind? Was it that students should be required to prosecute the study of medicine regularly and uninterruptedly? So far from it, students under the improved plan are actually forbidden to take a second course of lectures without an interval of some months. This is a change made with the ostensible object of improving the system of medical education, but really with no such motive, we think any sane man will conclude. It is in medicine, as in religion and politics; so long as we are contented with being tributaries, and tamely submit to the most unreasonable fanatical dogmas, all is well when these cease the war commences.

The tenacity with which the North adheres to the doctrine of Southern inequality in every particular and the degrading terms upon which we are to be permitted to enjoy their friendship, prove the utter hopelessness of fraternizing in a political union.

In view of all these things, we have no regrets for the threatened rupture of the political ties that bind us together, but rejoice in the hope that soon the present representaotin to the medical, as well as political Congress of the Union will be severed, firmly as has been our attachment to both, and rigidly as we have always adhered to the observance of their enactments.

Should such political separation occur—the consummation of which we ardently desire—the "M. A. M. A." would not be sought by southern physicians, as an appendix to their name; but it is not unreasonable to suppose, should there be a desire to change the M. D., for the indication of membership in an association, that M. S. C. M. A., or some such train of abreviations, might be more suitable to their taste, and more appropriate under the circumstances which surround us. We would then insist on retaining the M. D. at present, and suggest, respectfully, to those colleges and individual members of the profession who desire the change, that they shed no more crocodile tears over the debased conbition of the profession, nor make the system of medical education a pretext for the accomplishment of other objects, but take the matter to heart in good earnest, by adopting such plan as will insure the thorough preparation of all, hereafter, who are admitted to the degree of Doctor in Medicine, by *real tests* of qualification, before the degree is conferred.

